# Sexual dysfunction in male patients with obesity: is it still being overlooked?

**DOI:** 10.1007/s42000-025-00657-z

**Published:** 2025-04-22

**Authors:** Vahit Can Cavdar, Yagmur Izgi, Feray Akbas

**Affiliations:** University of Health Sciences, Department of Internal Medicine, Istanbul Training and Research Hospital, Istanbul, Turkey

**Keywords:** Obesity, Sex hormones, Aging males’ symptoms, International index of erectile function-5

## Abstract

**Introduction:**

Obesity has been linked to an elevated susceptibility to development of erectile dysfunction, yet the interplay between sex hormone levels, sexual function, and obesity remains unclear. This study aimed to investigate sexual dysfunction among male patients with obesity and to emphasize the importance of recognizing the problem and pursuing solutions.

**Methods:**

A total of 60 patients were included in the study (30 patients from the obesity center and 30 patients without obesity as the control group). Assessment of androgen hormone deficiency and erectile dysfunction was conducted through the implementation of AMS and IIEF-5 tests. The questionnaire includes aspects of medical history, demographic features, and lifestyle factors. Comprehensive measurements included BMI, WC, BP, lipid panel, total/free testosterone, sex-hormone-binding-globulin (SHBG), dehydroepiandrosterone-sulfate (DHEAS), fasting blood glucose (FBG), fasting insulin, and HbA1C levels. Results were evaluated using SPSS.

**Results:**

The AMS score in the obesity group was significantly lower compared to the group without obesity. The IIEF-5 score did not exhibit a statistically significant difference between the groups. Testosterone, free testosterone, SHBG, and HDL values were lower in the obesity group compared to the group without obesity.

**Conclusion:**

Although conducted in a small sample, our findings strongly indicate a positive correlation between obesity and the risk of moderate to severe ED. Most of the time, this condition goes unarticulated, thereby adversely affecting the quality of life for individuals with obesity. Clinicians should pay more attention to patients experiencing sexual dysfunction, especially those with obesity.

## Introduction

Obesity is a leading chronic disease that has evolved into a global pandemic over the last decade. Comorbidities and complications associated with the disease can be, moreover, extremely complex and serious [1].

Erectile dysfunction is one of the consequences of obesity. Erectile dysfunction, also referred to as impotence, is characterized by the persistent inability to achieve and sustain a penile erection of adequate rigidity for the facilitation of satisfactory sexual intercourse [2]. Beyond its impact on both the patient’s and the partner’s sexual experience, erectile dysfunction is also acknowledged as a potential indicator of underlying health issues in men [3].

The presence of erectile dysfunction is well-known to be linked to age and the presence of various comorbidities, including obesity, metabolic syndrome (MetS), diabetes, hypogonadism, and cardiovascular disease [4].

Drugs used to treat comorbidities related to obesity might also have an impact on sexual functions. Among antidiabetic agents metformin is the most studied, having beneficial effects on erectile dysfunction. GLP-1 RAs (glucagon-like peptide-1 receptor agonists) are also showing promising effects in studies; however, more studies are needed on acarbose, pioglitazone, DPP-4i (dipeptidyl-peptidase-4 inhibitors) and SGLT-2i (sodium-glucose cotransporter-2 inhibitors) [5].

The most reliable data sourced from the Massachusetts Male Aging Study revealed that the prevalence of minimal, moderate, and complete impotence was 52%, increasing with age. Projections indicate that by the year 2025, the global incidence of erectile dysfunction will surpass 300 million cases [6].

Obesity causes sexual dysfunction through a multifaceted array of pathways, mainly with a decline in sex hormone levels in men [7]. The reduction in sex hormone binding globulin (SHBG) levels, attributed to insulin resistance in individuals with obesity, has emerged as a primary factor in the decrease in testosterone levels. Another mechanism involves the inhibition of luteinizing hormone (LH) secretion through negative feedback, subsequent to the conversion of testosterone to estrogen, facilitated by the increased aromatase effect in adipose tissue. This cascade leads to diminished testosterone secretion. Furthermore, inflammatory cytokines released from adipose tissue impede testosterone release. Elevated levels of leptin disrupt the release of gonadotropins, precipitating hypogonadism. The resultant decrease in testosterone further exacerbates the accumulation of adipose tissue and the reduction of muscle tissue, creating a vicious circle [8].

Frequently, male patients experience feelings of embarrassment and shame, leading to the underreporting of male sexual dysfunction. Additionally, the issue remains unaddressed due to conservative cultural norms and religious beliefs. As a result, the consideration of obesity within the differential diagnosis of male sexual dysfunction may be inadvertently neglected [9].

Hence, it is essential to determine the prevalence of sexual dysfunction in individuals with obesity with the ultimate aim of highlighting this problem and ensuring the timely implementation of necessary interventions. There is thus a pressing need to encourage affected men to seek consultation at urology outpatient clinics.

This study aimed to investigate sexual dysfunction among male patients with obesity and to emphasize the importance of recognizing the problem and pursuing solutions.

## Materials and methods

The study was conducted at a teaching hospital in Istanbul, Turkiye, between June 2023 and January 2024. A case group of 30 patients with BMI ≥ 30 kg/m2 from the obesity center and a control group of 30 patients without obesity from the internal medicine outpatient clinic were included in the study after their consent was obtained in accordance with the inclusion and exclusion criteria.

The inclusion criteria were as follows: being ≥ 18 years of age, having a BMI ≥ 30 kg/m2 for the obesity group, and having a BMI < 30 kg/m2 for the control group. Exclusion criteria were as follows: the presence of diagnosed gonadal disease, nephrotic syndrome, cirrhosis, hypothyroidism, hyperthyroidism, HIV infection, glucocorticoid therapy, androgen replacement therapy, anticonvulsant therapy, active or previous history of malignancy, presence of acute inflammatory diseases, collagen vascular disorders, malnutrition-related disorders, and neurological disorders.

The medical history of the patients was obtained and age and gender were recorded. Weight, height, waist circumference (WC), and blood pressure were measured. Height was assessed without footwear to the nearest 0.5 cm, while body weight was measured to the nearest 0.1 kg with participants wearing light clothing and no shoes, utilizing electronic scales. WC was measured with gentle breathing at the midpoint between the lowest rib and the iliac crest, rounded to the nearest 0.1 cm with non-elastic tape. Body mass index (BMI) was calculated as weight (in kilograms) divided by the square of height (in meters). Systolic blood pressure (SBP) and diastolic blood pressure (DBP) were obtained in accordance with the World Health Organization definition. Participants, having sat quietly for 5 min, had their seated blood pressure measured three times by an internal medicine doctor using a sphygmomanometer. The average values of SBP and DBP were then calculated. Blood samples were drawn from the antecubital vein after a 12-hour fast and were stored at– 20 °C for subsequent hormone analysis. The analyses were conducted at the Medical Biochemistry Laboratory of the Istanbul Training and Research Hospital, University of Health Sciences. Total testosterone, free testosterone, sex hormone binding globulin (SHBG), dehydroepiandrosterone sulfate (DHEAS), and fasting insulin were measured with the chemiluminescence assay method using the Beckman Coulter DxI 800 immunoanalyser (Beckman Coulter Inc., CA, USA). Total cholesterol, TG, HDL, and LDL were measured with the photometric method using the AU 5800 autoanalyser (BeckmanCoulter Inc., CA, USA). HbA1c was measured with the high-performance liquid chromatography (HPLC) method using the Premier Hb9210 Analyzer (Trinity Biotech, Ireland).

AMS (aging male symptoms) and IIEF-5 (international index of erectile function) questionnaires were applied to the patients. An AMS score of 37 points and above was considered as abnormal in our study and IIEF-5 scores were evaluated in four categories, as follows: no erectile dysfunction (22–25 points), mild (17–21 points), moderate (8–16 points), and severe (5–7 points) erectile dysfunction.

Results were evaluated using SPSS 27.0 program.

### Statistical method

Quantitative variables are presented using mean, SD, median, min and max, while qualitative variables are presented using absolute and relative (%) frequencies. The distribution of variables was checked with the Kolmogorov-Simirnov test. The independent Samples t-test and Mann-Whitney U test were used for the comparison of quantitative data. The chi-square test was used for the comparison of the qualitative data. SPSS 27.0 (IBM Corp. Released 2020. IBM SPSS Statistics for Windows, Version 27.0. Armonk, NY: IBM Corp) was used for statistical analysis. The statistical significance level was considered to be *P* < 0.05.

## Results

In total, 60 patients, including 30 patients with obesity and 30 patients without obesity as the control group were included in the study. The mean age of the patients was 36.8 ± 9.5 years (38.8 ± 8.8 years in the obesity group and 34.8 ± 10 in the control group). The age of the obesity group was significantly higher compared to the group without obesity (*p* = 0.051). The parameters that were searched are listed in Table 1.

**Table 1:** Results of the parameters searched for the whole group

		Min-Max			Median	Mean ± sd/*n*-%		
Age		18.0	-	61.0	33.5	36.8	±	9.5
Height(cm)		164.0	-	201.0	179.0	179.3	±	7.8
Weight (kg)		50.0	-	126.0	90.5	94.6	±	17.5
BMI(kg/m²)		17.5	-	45.2	29.5	29.6	±	5.8
Medication	(-)					49		81.7%
	(+)					11		18.3%
AMS Score	Normal					46		76.7%
	Pathological					14		23.3%
AMS Score		20.0	-	65.0	31.0	32.5	±	8.9
IIEF-5 Score	No E.D (22–25)					19		31.7%
	Severe E.D (5–7)					1		1.7%
	Moderate E.D (8–16)					13		21.7%
	Mild E.D (17–21)					27		45.0%
IIEF-5 Score		7.0	-	25.0	21.0	19.1	±	4.3
WC (cm)		78.0	-	154.0	100.0	104.9	±	16.9
T.Testosterone(µg/L)		1.8	-	10.5	4.6	4.8	±	2.1
F.Testosterone(pg/ml)		6.8	-	51.9	23.8	25.8	±	10.4
SHBG(nmol/L)		8.2	-	74.5	25.7	28.5	±	13.0
DHEAS(µg/dL)		68.0	-	613.0	277.0	299.0	±	121.4
FBG(mg/dL)		68.0	-	187.0	91.0	96.4	±	22.7
Insulin(mg/dL)		1.3	-	32.1	9.3	11.2	±	7.4
HbA1c(%)		4.7	-	9.3	5.8	5.9	±	0.9
T.cholesterol(mg/dL)		86.0	-	318.0	186.0	191.3	±	47.6
LDL(mg/dL)		42.0	-	223.0	113.0	113.3	±	34.2
TG(mg/dL)		49.0	-	614.0	137.0	178.1	±	138.0
HDL(mg/dL)		28.0	-	72.0	40.0	41.8	±	9.7

**Fig. 1:**
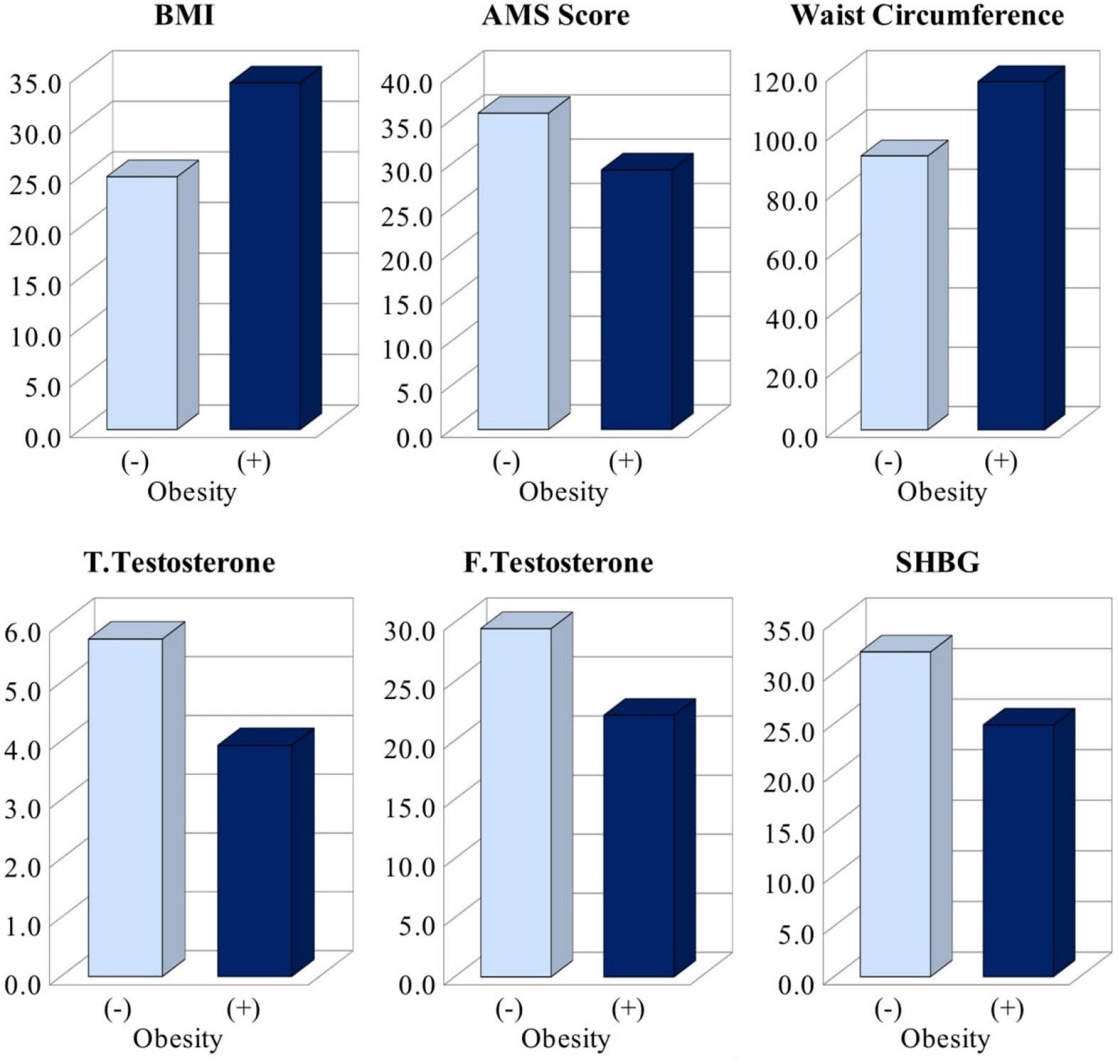
Comparison of results for the obesity and control groups

Comparison of results for the obesity and the control group are shown in Table 2; Fig. 1. The mean BMI of patients was 29.6 ± 5.8 kg/m2 (34.2 ± 4.0 kg/m2 in the obesity group and 25 ± 2.8 kg/m2 in the control group). Both weight (*p* < 0.001) and BMI (*p* < 0.001) values were higher in the obesity group compared to the control group. There was no statistically significant difference in height between the obesity group and the control group (*p* = 0.215). The mean WC was 104.9 ± 16.9 cm (117.4 ± 14.3 in the obesity group and 92.3 ± 7.2 cm in the control group). WC was higher in the obesity group compared to the control group (*p* < 0.001) (Table 2).

**Table 2:** Comparison of results for the obesity and control groups

		Obesity (-)					Obesity (+)				*p*	
		Mean ± sd/*n*-%			Median		Mean ± sd/*n*-%			Median		
Age		34.8	±	10.0	32.0		38.8	±	8.8	38.5	***0.051***	^m^
Height(cm)		180.6	±	7.3	181.5		178.1	±	8.2	178.0	0.215	^t^
Weight (kg)		81.1	±	9.9	81.5		108.1	±	12.2	108.0	***0.000***	^t^
BMI(kg/m²)		25.0	±	2.8	25.5		34.2	±	4.0	32.8	***0.000***	^m^
Medication	(-)	28		93.3%			21		70.0%		***0.020***	^X²^
	(+)	2		6.7%			9		30.0%			
AMS Score	Normal	18		60.0%			28		93.3%		***0.002***	^X²^
	Pathological	12		40.0%			2		6.7%			
AMS Score		35.7	±	9.6	35.0		29.3	±	6.9	28.5	***0.002***	^m^
***IIEF-5 Score***												
No E.D (22–25)		10		33.3%			9		30.0%		0.781	^X²^
Severe E.D (5–7)		1		3.3%			0		0.0%			
Moderate E.D (8–16)		6		20.0%			7		23.3%			
Mild E.D (17–21)		13		43.3%			14		46.7%			
IIEF-5 Score		19.1	±	4.3	21.0		19.1	±	4.4	21.0	0.964	^m^
WC (cm)		92.3	±	7.2	93.5		117.4	±	14.3	118.0	***0.000***	^t^
T.Testosterone(µg/L)		5.7	±	2.3	5.2		3.9	±	1.5	3.3	***0.002***	^m^
F.Testosterone(pg/ml)		29.5	±	11.5	25.6		22.1	±	7.8	20.5	***0.008***	^m^
SHBG(nmol/L)		32.1	±	13.4	28.9		24.9	±	11.7	22.1	***0.018***	^m^
DHEAS(µg/dL)		301.5	±	107.3	295.5		296.5	±	135.8	267.0	0.876	^t^
FBG(mg/dL)		91.7	±	20.4	88.0		101.0	±	24.2	93.5	***0.041***	^m^
Insulin(mg/dL)		7.4	±	5.4	6.1		15.0	±	7.3	14.4	***0.000***	^m^
HbA1c(%)		5.9	±	0.5	5.9		6.0	±	1.2	5.7	0.578	^m^
T.cholesterol(mg/dL)		178.2	±	45.2	179.5		204.4	±	47.0	193.0	***0.024***	^m^
LDL(mg/dL)		107.0	±	31.7	112.0		119.5	±	36.0	115.5	0.161	^t^
TG(mg/dL)		132.9	±	93.8	112.5		223.2	±	160.4	162.0	***0.003***	^m^
HDL(mg/dL)		44.7	±	10.6	43.0		38.8	±	7.9	36.5	***0.017***	^t^

^t^ Independent samples t-test / ^m^ Mann-Whitney U test / ^X²^ Chi-square test

A total of 18.3% (11 patients) of the patients were using medication due to comorbid diseases. 30% (nine patients) in the obesity group and 6.7% (two patients) of the patients in the control group were using medication due to comorbid diseases. The prevalence of drug treatment was higher in the obesity group compared to the control group (*p* = 0.020) (Table 2).

Biochemical parameters that were searched are listed in Table 2. The obesity group had higher levels of FBG (*p* = 0.041), insulin (*p* < 0.001), total cholesterol (*p* = 0.024), and TG (*p* = 0.003) values compared to the control group. HDL values were lower in the obesity group when compared to the control group (*p* = 0.017). HbA1c (*p* = 0.578) and LDL (*p* = 0.161) values showed no significant differences between the two groups.

Sex hormone results are listed in Table 2. Total testosterone (*p* = 0.002), free testosterone (*p* = 0.008) and SHBG (*p* = 0.018), values were lower in the obesity group when compared to the control group. DHEAS levels showed no significant difference between the groups (*p* = 0.876).

The mean AMS score was 29.3 ± 6.9 in the obesity group and 35.7 ± 9.6 points in the control group. In total, 23.3% (14 patients) of the patients had abnormal AMS scores. The mean AMS score was found to be abnormal in 6.7% (two patients) of the patients in the obesity group and 40% (12 patients) of the patients in the control group. The AMS score was significantly lower in the obesity group when compared to the control group (*p* = 0.002).

According to the IIEF-5 score the results were the following: In the obesity group, no erectile dysfunction was detected in 30% (nine patients) of the patients, mild erectile dysfunction was detected in 46.7% (14 patients) of the patients, moderate erectile dysfunction was detected in 23.3% (seven patients), and severe erectile dysfunction was not detected in any patients. In the control group; there was no erectile dysfunction in 33.3% (ten patients) of the patients, mild erectile dysfunction in 43.3% (13 patients) of the patients, moderate erectile dysfunction in 20% (six patients) of the patients, and severe erectile dysfunction in 3.3% (one patient) of the patients. The IIEF-5 score did not exhibit a statistically significant difference between the groups (*p* = 0.781) (Table 2).

## Discussion

The underlying causes of obesity are widely recognized globally, including genetic, environmental, socioeconomic, and behavioral factors, as supported by the literature [10]. While genetic predisposition is often considered a significant factor in weight gain, studies have indicated that it does not solely determine the development of obesity. Instead, factors such as dietary habits and physical activity play a more crucial role in weight gain [11–12].

Obesity is associated with a spectrum of health issues, including type 2 diabetes, prediabetes, cardiovascular diseases, hypertension, hyperlipidemia, cerebrovascular disease, various cancers, obstructive sleep apnea syndrome, non-alcoholic fatty liver, infertility, and depression [13–21]. Given that obesity frequently coincides with numerous comorbid conditions, the treatment of patients with obesity necessitates the administration of effective drugs with diverse mechanisms [22]. In our study, 30% of the case group and 6.7% of the control group were taking medications for comorbid diseases, supporting the above data.

In our study, a comparison between the obesity group and the control group revealed significantly elevated levels of fasting blood sugar, fasting insulin, total cholesterol, and triglycerides in the obesity group, aligning with findings from previous research [13, 15, 23]. These outcomes demonstrate the strong relationship between obesity and its associated metabolic disorders. Establishing causality and underlying mechanisms remains challenging, as erectile dysfunction and metabolic conditions share common risk factors, including age, diabetes, hypertension, insulin resistance, smoking, and increased body mass index [24]. Consequently, as in our study, it is difficult to determine the extent to which erectile dysfunction is attributable to specific elements of accompanying cardiometabolic or endocrine conditions.

Erectile dysfunction can also be an early marker for glycemic or endocrine disorders. In a study Mazzilli et al., 30% of the patients who were diagnosed with erectile dysfunction were found to have undiagnosed endocrine and glycemic disorders, such as diabetes, prediabetes, hypogonadism, thyroid dysfunction, and hyperprolactinemia [25]. Furthermore, erectile dysfunction can be a sign of present or future cardiovascular events [26]. Thus, in addition to diagnosed conditions, potential comorbidities should also be screened following the initial diagnosis. Since our patients were recruited from an obesity center, they were all screened for these disorders.

Research has consistently demonstrated that obesity causes a decrease in sex hormone levels in men through several mechanisms. In cases of moderate obesity, the reduction in total testosterone is attributed to the decrease in SHBG, a consequence of insulin resistance. In instances of severe obesity, the suppression of the hypothalamic-pituitary-testicular axis, coupled with the mechanism involving insulin resistance, further leads to a decrease in free testosterone levels [27, 28]. Numerous studies demonstrate that patients with obesity exhibit low levels of total testosterone, free testosterone, and SHBG [29, 30]. Our study also revealed a statistically significant decrease in total testosterone, free testosterone, and SHBG levels in the obesity group compared to the control group, indicating a significant decrease in these hormonal parameters in the obesity group.

The underlying mechanisms of sexual dysfunction in male individuals with obesity, other than hormonal imbalance and insulin resistance, include endothelial dysfunction, psychological factors, and physical inactivity [31].

Different questionnaires and scales are applied to assess sexual dysfunction and erectile dysfunction in men: in our study we utilized the AMS and IIEF-5 questionnaires.

The AMS scale, of high value and reliability and utilized worldwide, is used to evaluate the health-related quality of life in aging men. This scale is standardized according to psychometric norms, making it a reliable tool for assessment [32]. Consistent with the scoring approach in the AMS study by Friedemann Zengerling et al., we accepted an AMS score of 37 points and above as abnormal in our study [33].

The International Index of Erectile Function (IIEF-5) is a trusted and validated tool for diagnosis of erectile dysfunction [34]. The IIEF-5 is a simplified version of the IIEF-15, consisting of five items. The first four items assess erectile function, while the fifth item questions satisfaction with intercourse [35]. In the questionnaire, Item 1 assesses the patient’s confidence in achieving and maintaining an erection, rated on a Likert-type scale from 1 to 5. Items 2 to 5 inquire about the patient’s experiences in the previous weeks. If the target population includes individuals who have had recent sexual intercourse, these items can be scored from 1 to 5 [35, 36]. Regarding the IIEF-5 score data, divergent classifications have been used by different researchers. In the classification made by Rhoden et al. [37], while the classification system as severe, moderate, mild-moderate, mild, and no erectile dysfunction was used, we utilized a classification system with four groups, namely, no erectile dysfunction (22–25 points), mild (17–21 points), moderate (8–16 points), and severe (5–7 points) erectile dysfunction [4] to allow evaluation of more subjects in each group.

We detected mild erectile dysfunction in 14 patients (46.7%) and moderate erectile dysfunction in seven patients (23.3%) in the obesity group based on the IIEF-5 score, along with the detection of erectile dysfunction in two patients (6.7%), according to the AMS score. These findings further substantiate the relationship between obesity and erectile dysfunction [2, 27, 38].

Certain tests employed for evaluating erectile dysfunction may not consistently demonstrate sufficient sensitivity and specificity in identifying androgen deficiency. A prior study involving 339 middle-aged Taiwanese men established that both the AMS scale and the ADAM questionnaire lacked the requisite sensitivity and specificity for detecting androgen deficiency [39]. The research highlighted a lack of a significant relationship between the total AMS score and testosterone or SHBG levels, indicating potential high sensitivity in detecting signs and symptoms of late-onset hypogonadism but comparatively weaker specificity [40]. In our study, there was no significant difference in IIEF-5 score between case and control groups. Contrary to expectations, a pathological AMS score was identified in only two patients (6.7%) in the obesity group, whereas 12 patients (40%) in the group without obesity exhibited a pathological AMS score. This observation aligns with the findings of the Taiwanese study, suggesting a discrepancy in the specificity of the AMS score in the context of obesity [39]. However, the possibility of disguising the problem due to personal or psychological reasons, given the subjective nature of these questionnaires, is also a concern. It is crucial to address all aspects of obesity in disease management and to help patients discuss related health issues, including sexual dysfunction, in a professional and non-stigmatizing manner.

## Conclusion

Sexual dysfunction has been known for some time to be a concurrent condition with obesity, often remaining, however, undisclosed due to feelings of shame and anxiety and significantly impacting the quality of life of individuals with obesity. It is important to investigate the prevalence of sexual dysfunction in this population thereby drawing attention to the issue and ensuring timely interventions.

Both existing studies and our own research emphasize the limitations of survey tests in terms of sensitivity and specificity for detection of low sex hormone levels in men, particularly in the context of sexual dysfunction and erectile dysfunction. A comprehensive clinical approach is necessary to effectively address sexual function in this patient population.

## Study limitations

The AMS and IIEF-5 surveys are subjective evaluations. The number of patients included in the study is limited and most patients in the obesity group fall within the stage 1 and stage 2 obesity categories, with BMI values similar to those in the control group. Patients with diabetes or hypertension were not excluded in both case and control groups, which means that these conditions or their medications could contribute to erectile dysfunction.

## Data Availability

Data are available upon request.
